# ^18^F-FDG/PET-CT imaging findings after sternotomy

**DOI:** 10.1007/s12350-022-03126-x

**Published:** 2022-11-08

**Authors:** Maurits S. H. Blomjous, Ties A. Mulders, Ali R. Wahadat, Wilco Tanis, Ad J. J. C. Bogers, Jolien W. Roos-Hesselink, Ricardo P. J. Budde

**Affiliations:** 1 Department of Radiology and Nuclear Medicine, Erasmus Medical Center, ND-547, Dr. Molewaterplein 40, 015GD Rotterdam, The Netherlands; 2 Department of Cardiology, Thoraxcenter, Erasmus Medical Center, Rotterdam, The Netherlands; 3 Department of Cardiology, HAGA Hospital, The Hague, The Netherlands; 4 Department of Cardiothoracic Surgery, Thoraxcenter, Erasmus Medical Center, Rotterdam, The Netherlands

## Abstract

**Background:**

The clinical diagnosis of deep sternal wound infection (DSWI) is supported by imaging findings including 18F-fluorodeoxyglucose positron emission tomography/computed tomography (^18^F-FDG-PET/CT). To avoid misinterpretation due to normal post-surgery inflammation we assessed normal imaging findings in non-infected patients after sternotomy.

**Methods:**

This is a prospective cohort study including non-infectious patients with sternotomy. All patients underwent ^18^F-FDG-PET/CT at either 5 weeks (group 1), 12 weeks (group 2) or 52 weeks (group 3) post-surgery. ^18^F-FDG uptake was scored visually in five categories and assessed quantitatively.

**Results:**

A total of 44 patients were included. Sternal mean SUVmax was 7.34 (± 1.86), 5.22 (± 2.55) and 3.20 (± 1.80) in group 1, 2 and 3, respectively (*p* < 0.01). Sternal mean SUVmean was 3.84 (± 1.00), 2.69 (± 1.32) and 1.71 (± 0.98) in group 1, 2 and 3 (*p* < 0.01). All patients in group 1 had elevated uptake whereas group 2 and 3 showed 2/15 (13%) and 11/20 (55%) patients respectively with no elevated uptake. Group 3 still showed an elevated uptake pattern in in 9/20 (45%) and in 3/9 (33%) with a high-grade diffuse uptake pattern.

**Conclusion:**

This study shows significant lower sternal ^18^F-FDG at 55 weeks compared to 5 weeks post-sternotomy however elevated uptake patterns may persist.

**Supplementary Information:**

The online version contains supplementary material available at 10.1007/s12350-022-03126-x.

## Introduction

Deep sternal wound infection (DSWI) occurs with an incidence of 1.8% following sternotomy for cardiac surgery and is associated with an increased risk of in-hospital mortality of 14.2%.^1^ Prolonged hospital stay due to DSWI is associated with postoperative morbidity such as renal failure, respiratory failure and stroke.^1^ Early recognition and treatment improve clinical outcome. According to guidelines from the Centers for Disease Control and Prevention, DSWI is diagnosed by either (I) positive local culture result; (II) evidence of mediastinitis seen during surgery; or (III) one of the following conditions: chest pain, sternal instability, or fever (> 38 °C) in combination with either purulent discharge from the mediastinum or a positive blood culture.^2^ The clinical diagnosis is supported by laboratory results and imaging findings. Imaging is particularly helpful in discriminating DSWI from superficial sternal wound infection which requires a different management strategy (3). Furthermore, imaging plays an important role in visualizing the anatomical extent of the infection and guide adequate debridement. ^18^F-fluorodeoxyglucose positron emission tomography/computed tomography (^18^F-FDG PET/CT) has increasingly been used over the last decade to detect inflammatory, infectious or malignant processes. Although literature addressing the utility of ^18^F-FDG PET/CT in patients with suspected DSWI after sternotomy is limited, smaller studies indicate promising accuracy.^4,5^

However, normal post-operative inflammatory changes might cause ^18^F-FDG uptake that could falsely be interpreted as pathological uptake and therefore limit the specificity of ^18^F-FDG PET/CT especially early after sternotomy. Misinterpretation of ^18^F-FDG PET/CT in patients with suspected DSWI can have severe therapeutic and prognostic consequences. To improve diagnostic accuracy, it is important that cardiologists, cardiothoracic surgeons, radiologists and nuclear medicine physicians gain further understanding of normal ^18^F-FDG evolution and sternal distribution patterns in patients after sternotomy without infection to distinguish normal from pathological uptake. We assessed sternal ^18^F-FDG uptake visually and quantitatively at three different time points within the first-year post-cardiothoracic surgery to describe normal ^18^F-FDG distribution patterns.

## Methods

### Study population

This is a secondary data analysis of two prospective cross-sectional studies that investigated normal ^18^F-FDG uptake around aortic valve prostheses and grafts.^6,7^. The medical ethics committee approved these studies (NL42743.041.12). Imaging studies performed at the Erasmus Medical Center, Rotterdam were used for analysis. Common eligibility criteria included patients aged 50 years or older, weight of < 110 kg, with no signs of endocarditis that had undergone sternotomy for an uncomplicated aortic valve replacement (AVR), supracoronary aortic replacement with AVR or aortic root replacement with Bentall prosthesis. Since this is a secondary data analysis, exclusion criteria were similar to the previous studies and included diabetes mellitus, left ventricular ejection fraction < 45%, active cardiac decompensation, uncontrolled cardiac arrhythmias, suspicion of endocarditis, previous participation in scientific studies using radiation, (possible) pregnancy in pre-menopausal women above 50 years not on reliable birth control therapy, use of pericardial patches, re-operation and refusal to be informed about potential FDG-PET findings.^6,7^ An uncomplicated procedure was defined as a procedure with no complications during or directly after surgery. All patients underwent ^18^ FDG PET-CT at either 5 weeks ± 1 (group 1), 12 weeks ± 2 (group 2) or 52 weeks ± 8 (group 3) post-surgery.

### ^18^F-FDG PET/CT image acquisition

**Figure 1:**
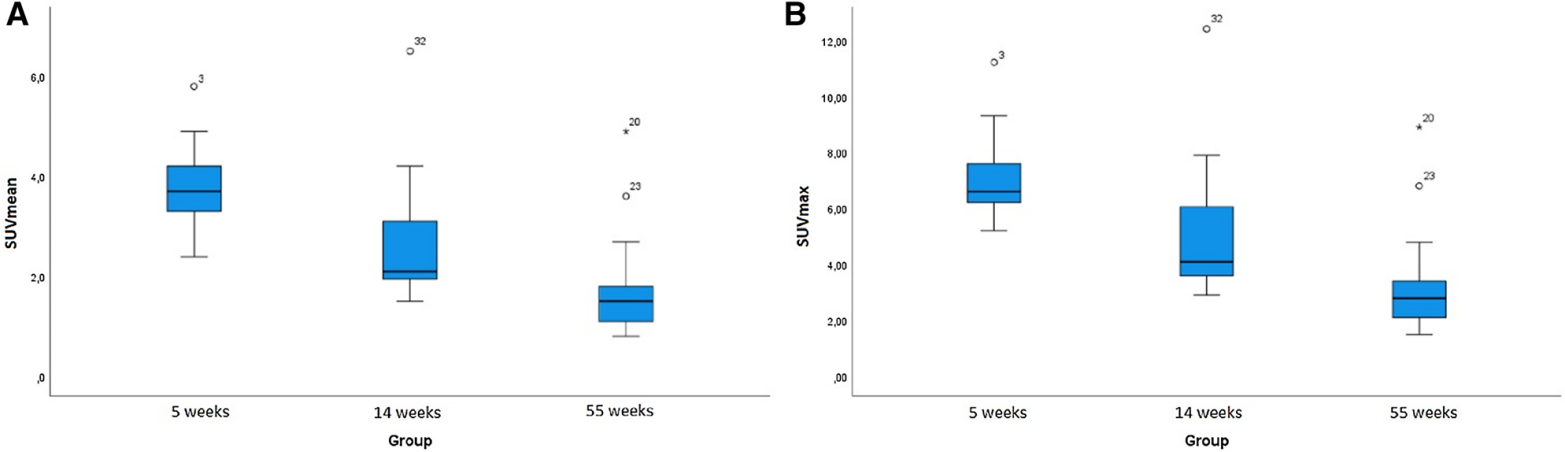
Boxplot of the SUVmean (A) and SUVmax (B) measurements distribution in each group. The dots indicated as “3”, “32”, “20”, “23”, (A, B) are outliers in the SUVmax and SUVmean measurements

Imaging acquisition protocols have been described in detail before.^6,7^ In brief, patients followed a 24-h low carbohydrate diet, of which the last 12 h were spent fasting to induce free fatty acid metabolism and suppress myocardial glucose metabolism. Thereafter, patients received an intravenous ^18^F-FDG-injection of 2.0 MBq/kg and were hydrated with 1000 ml of water 1 h prior to image acquisition. Blood glucose levels were checked before ^18^F-FDG injection and the limit was set to 8.9 mmol/l. Approximately 1 h after ^18^F-FDG injection, the PET/CT was performed using an EANM Research Ltd. (EARL) accredited Biograph Sensation 16scanner (SIEMENS Medical, Germany). Before the PET acquisition, a low dose CT-scan was performed for attenuation correction. A more detailed PET-scan of the heart was then obtained with 3-min acquisitions per bed position using a 3-dimensional acquisition mode. Attenuation corrected PET images were reconstructed with an ordered-subset expectation–maximization iterative reconstruction algorithm (Fig. 1).

### Image analysis and interpretation

**Figure 2:**
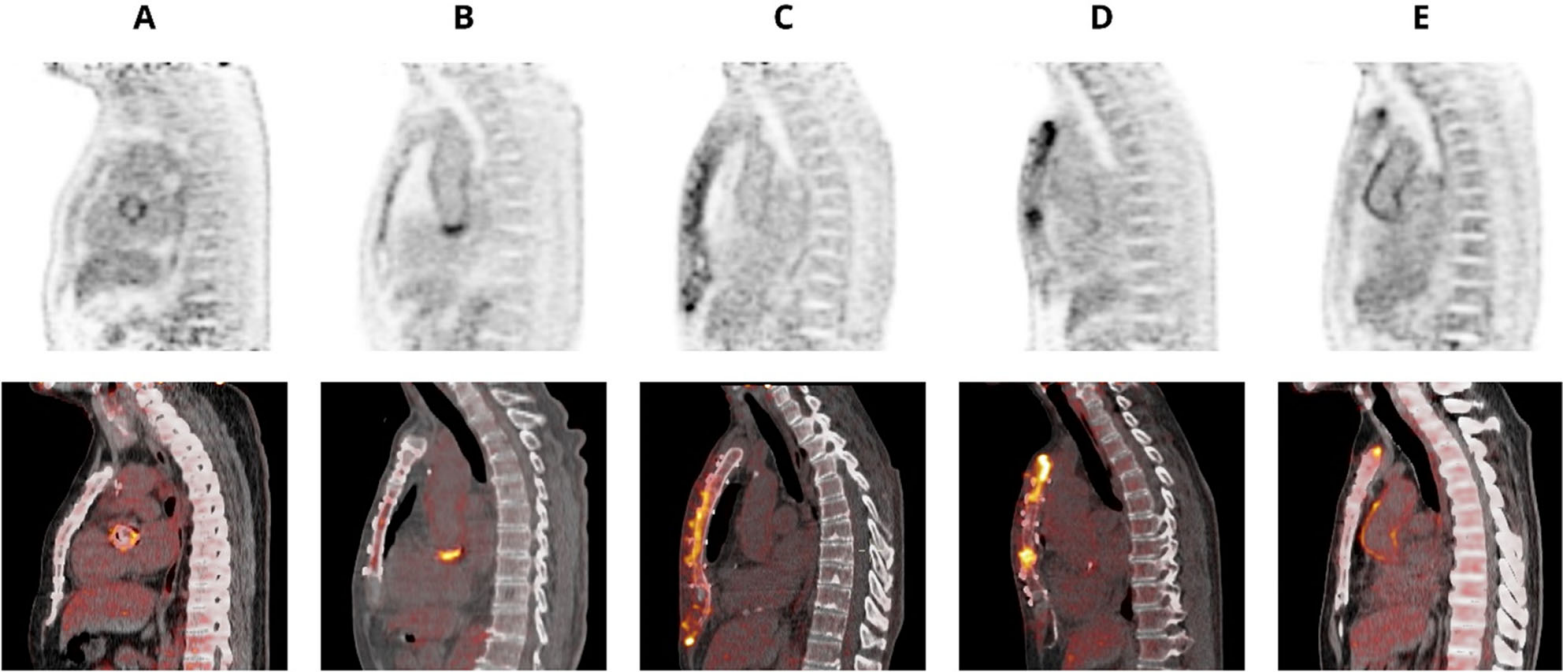
Examples of different categories of sternal ^18^FDG uptake patterns post-sternotomy. (A) no elevated uptake; (B) low-grade diffuse uptake; (C) high-grade diffuse uptake (D); high-grade heterogeneous uptake; (E) focal uptake

Uptake of ^18^F-FDG was scored visually and quantitatively for all patients by an experienced nuclear medicine physician (TM). Visually, sternal ^18^F-FDG uptake was scored as one of five different categories: A: No elevated uptake; B: Low-grade diffuse uptake; C: High-grade diffuse uptake; D: High-grade heterogeneous uptake; V: Focal uptake. Examples of different categories are shown in Fig. 2.

**Figure 3:**
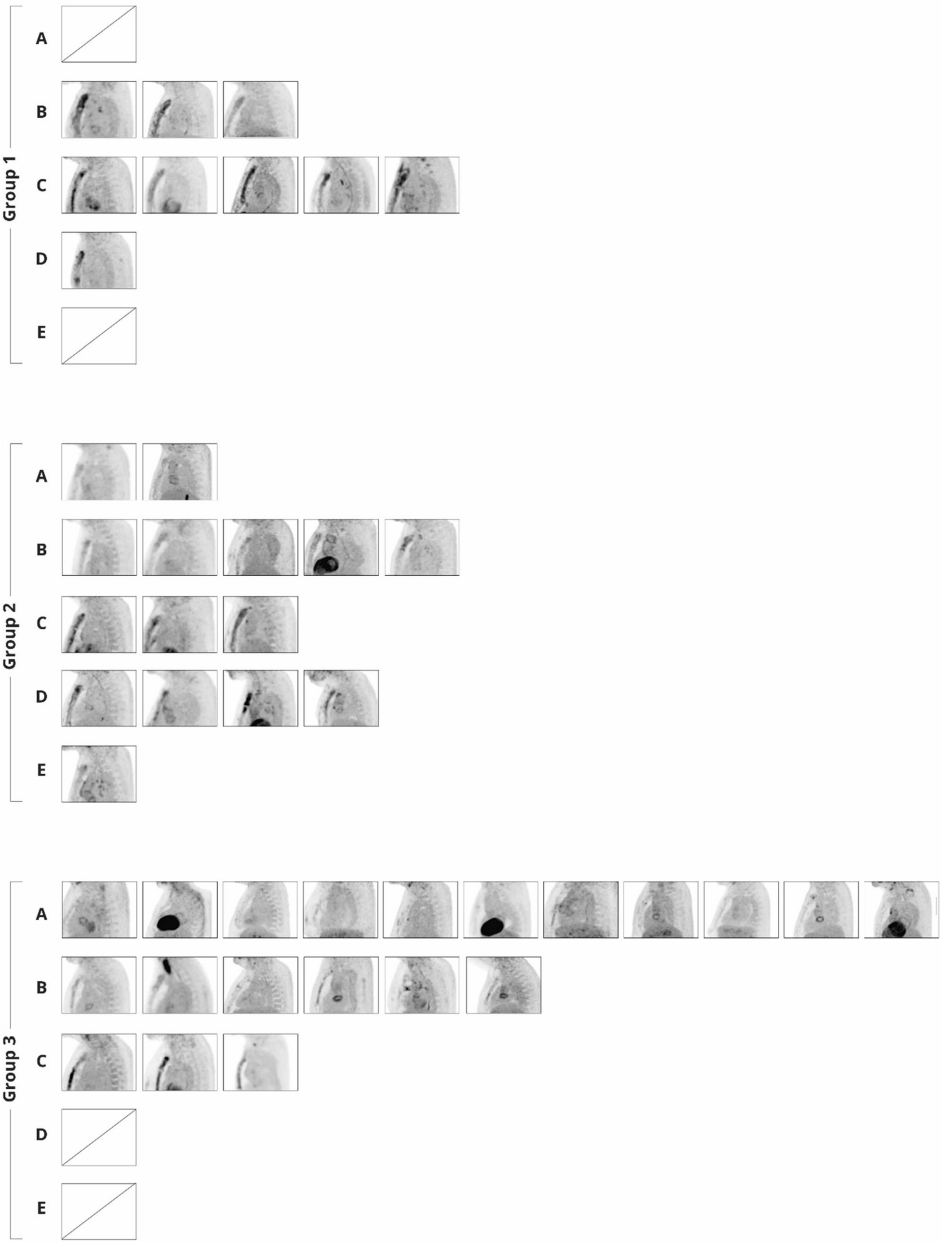
Sternal ^18^F-FDG uptake on lateral view of attenuation-corrected (AC) maximum intensity projection (MIP) images in all patients divided by group and subdivided by visual uptake score. Group 1 (5 weeks post-sternomy); Group 2 (14 weeks post-sternomy); Group 3 (55 weeks post-sternomy). Category (A); no eleveated uptake; Category (B) low-grade diffuse uptake; Category (C) high-grade diffues uptake; Category (D) High-grade heterogeneous uptake; Category (E) focal uptake

Quantitative analysis consisted of measuring Maximum Standardized Uptake Value (SUV_max_) and Mean Standardized Uptake Value (SUV_mean_) using commercially available software (Carestream v12.2.2.1025, Rochester, United States of America). SUV_max_ was measured in an automated volume of interest (VOI) at the region with the highest uptake in the sternum by visual assessment. The SUV_mean_ was measured in a VOI including the whole sternum using manual and automatic segmentation. The SUV_mean_ of the bloodpool was measured in the descending aorta, taking care not to include the vessel wall.

Additional findings that were scored were presence of: A: Lymph nodes with elevated uptake in the mediastinum, pulmonary hilum, parasternal side and axilla; B: Pericardial effusion with or without elevated uptake; C: Retrosternal fluid collection, with or without elevated uptake; D: Subcutaneous uptake at the sternotomy scar; E: Complete ossification of the sternum.

### Statistics

Analyses were performed using SPSS (version 25.0; SPSS Inc.). Descriptive statistics were used for analysis of the outcomes. For continuous variables, means and standard deviations (SD) were used in case of normal distribution. In case of non-normal distribution, medians and interquartile ranges (IQR) were used. The IQR and confidence interval (CI) were denoted in square brackets. Comparison between more than two groups was made using the one-way ANOVA test for normal distributed continuous variables and Kruskal–Wallis test for non-normal distributed continuous variables. Dunn’s pairwise test was used for group comparison in the post hoc analysis. Chi-square test was used for categorical variables. A significance level of *p* = 0.05 and 95% confidence intervals (CI) were used.

## Results

### Patient characteristics and classification

A total of 44 patients were included of which n = 9 in group 1, n = 15 in group 2 and n = 20 in group 3. Combining data from two prospective cross-sectional studies resulted in the unequal group sizes. Age (mean ± SD) at time of imaging was 64 ± 7 years and most patients were male 29/44 (65%). No patient was clinically suspected of DSWI at the time of performing ^18^F-FDG PET/CT. Baseline characteristics for the overall population and the three groups are summarized in Table 1.

**Table 1:** Baseline characteristics of all patients and for each patient per group

	All included patients	Group I	Group 2	Group 3	P-value**
		(5 weeks (± 1) post-surgery)	(14 weeks (± 2) post-surgery)	(55 weeks (± 5) post-surgery)	
Number of patients, n	44	9	15	20	
Age, mean ± SD, years	64 ± 7.0	63 ± 6.1	66 ± 7	63 ± 7	0.35
Days between PET-CT and sternotomy, median [IQR]	117 [87–371]	36 [33–38]	92 [88–96]	373 [356–414]	< 0.01
*Gender*, n(%)					
Male, n(%)	29 (66)	6 (67)	11 (73)	12 (60)	0.37
Female, n(%)	15 (34)	3 (33)	4 (27)	8 (40)	
BSA, (mean ± SD)	1.97 ± 0.21	1.89 ± 0.20	1.92 ± 0.18	2.03 ± 0.22	0.15
Length, cm (mean ± SD)	174 ± 12	172 ± 12	173 ± 13	175 ± 11	0.86
Weight, kg (mean ± SD)	81 ± 14	77 ± 13	78 ± 11	87 ± 15	0.07
Laboratory results*					
Serum levels of leucocytes × 10^9^/L, mean ± SD	10.64 ± 0.38	10.2 ± 0.92	10.76 ± 0.67	10.75 ± 0.55	0.87
Serum levels of glucose mmol/L, mean ± SD	5.2 ± 0.09	5.4 ± 0.15	5.71 ± 0.16	5.25 ± 0.16	0.12
Serum levels of creatinine μmmol/L, mean ± SD	75.81 ± 2.72	77 ± 7.56	78.33 ± 4.49	73.4 ± 3.76	0.71

*SD,* standard deviation; *IQR,* interquartile range; *PET-CT;* positron emission tomography with computed tomography

*Serum Leucocytes and creatinine levels were measured as part of clinical practice ± 5 days after surgery and glucose levels were measured on the day of 18-FDG PET-CT scan

** Statistical difference between the three groups 1, 2 and 3

### ^18^F-FDG PET-CT findings

The median time between sternotomy and ^18^F-FDG PET/CT was 5 ± 1 weeks (group 1), 14 ± 2 weeks (group 2) or 55 ± 5 weeks for group 1, 2 and 3, respectively. It was not always possible to scan in the planned time window causing actual time between surgery and imaging to deviate from the planned duration. Median ^18^F-FDG dosage was 168 MBq/kg and not significant different between the three groups (*p* = 0.10). One patient showed pulmonary infection as an incidental finding which resolved on follow up imaging.

The mean ± SD sternal SUV_max_ was 7.34 ± 1.86, 5.22 ± 2.55 and 3.20 ± 1.80 in group 1, 2 and 3, respectively (*p* < 0.01). The mean ± SD of sternal SUV_mean_ was 3.84 ± 1.00, 2.69 ± 1.32 and 1.71 ± 0.98 in group 1, 2 and 3 (*p* < 0.01) (Fig. 1). Pairwise comparison of groups in the post hoc analysis showed no significant difference between 5 weeks post-sternotomy and 14 weeks sternotomy (group 1 vs. 2). All other groups relative to each showed statistical difference to each other for both SUV_max_ and SUV_mean_ (*p* = 0.07 and *p* = 0.09, respectively).

Figure 3 presents an overview of sternal FDG activity in all 44 patients divided by group and visual uptake category. None of the patients in group 1 showed an uptake category A, whereas in group 2 and 3 2/15 (13%) and 11/20 (55%) respectively, showed uptake category A. Group 1 showed uptake category C in 5/9 (56%) which was less often observed in group 2 and 3 with 3/15 (20%) and 3/20 (15%) patients, respectively. Group 3 showed an elevated uptake pattern (either category B, C, D or E) in 9/20 (45%), caused by category B in 6/9 (66%) and category C in 3/9 (33%) of the patients.

Subcutaneous elevated uptake along the sternotomy scar was observed in n = 9/9 (100%), n = 13/15 (87%) and 9/20 (45%) patients in group 1, 2 and 3, respectively (0.01). Complete ossification of the sternum was observed in n = 0/9 (0%), n = 5/15 (33%) and n = 10/20 (50%) patients, respectively in group 1, 2 and 3 (*p* < 0.01). With respect to visual uptake patterns complete ossification was present only in categories A and B and not in categories C, D and E (*p* < 0.01). Mediastinal, hilar, parasternal and axillary lymph nodes with elevated uptake were seen in n = 9 (20%), n = 6 (14%), n = 2 (5%) and n = 3 (7%), respectively. Pericardial effusion was seen in n = 5 (11%) patients with elevated uptake in n = 2 (5%) patients. Retrosternal fluid collection occurred in n = 1 (5%) and showed elevated uptake. There was no significant difference in elevated lymph node uptake (mediastinal, hilar, parasternal and axillary), pericardial effusion (*p* = 0.09) and retrosternal fluid collections ( 0.14) in comparison between groups. All ^18^F-FDG PET-CT findings are summarized in Table 2.

**Table 2:** ^18^F-FDG PET-CT findings for all patient and for each patient per group

	All included patients	Group 1	Group 2	Group 3	*P*-value**
		(5 weeks (± 1) post-surgery	(14 weeks (± 2) post-surgery)	(55 weeks (± 5) post-surgery)	
Number of patients, n	44	9	15	20	
^18^F-FDG dose, MBq/kg, median [IQR]	168 [146–185]	153 [132–175]	157[145–175]	182 [161–192]	0.10
Time between FDG dose and start scan (min), median [IQR]	59 [57—62]	59 [56–62]	60 [57–65]	59 [57–61]	0.49
SUVmax bloodpool	2.00 ± 0.23	1.91 ± 0.22	2.01 ± 0.25	2.05 ± 0.23	0.40
SUVmax sternum	4.73 ± 2.61	7.34 ± 1.86	5.22 ± 2.55	3.20 ± 1.80	< 0.01
SUVmean sternum	2.80 ± 1.37	3.84 ± 1.00	2.69 ± 1.32	1.71 ± 0.98	< 0.01
*Visual analysis uptake sternum*					0.01
Category A, n(%)	13 (30)	0	2 (13)	11 (55)	
Category B, n(%)	14 (32)	3 (33)	5 (33)	6 (30)	
Category C, n(%)	5 (11)	1 (11)	4 (27)	0	
Category D, n(%)	11 (25)	5 (56)	3 (20)	3 (15)	
Category E, n(%)	1 (5)	0	1 (7)	0	
*Elevated uptake lymph node*					
Mediastinal, n(%)	9 (20)	3 (33)	3 (20)	3 (15)	0.53
Hilar, n(%)	6 (14)	0	2 (13)	4 (20)	0.35
Parasternal, n(%)	2 (5)	1 (11)	1 (20)	0	0.37
Axillary, n(%)	3 (7)	0	2 (13)	1 (5)	0.41
Pericardial effusion, n(%)	5 (11)	2 (22)	3 (20)	0	0.09
with elevated uptake, n(%)	2 (5)	1 (11)	1 (7)	0	0.37
Retrosternal fluid collection, n(%)	1 (5)	1 (11)	0	0	0.14
with elevated peripheral uptake, n(%)	1 (5)	1 (11)	0	0	0.20
Elevated uptake scar, n(%)	31 (70)	9 (100)	13 (87)	9 (45)	< 0.01
Complete ossification sternum, n(%)	15 (34)	0	5 (33)	10 (50)	< 0.01

SUVmax: Maximum standardized uptake value; standardized SD: Standard deviation; IQR, interquartile range; PET-CT; positron emission tomography with computed tomography. Category A; no elevated uptake; Category (B) low-grade diffuse uptake; Category (C) high-grade diffuse uptake; Category (D) high grade heterogeneous uptake; Category (E) focal uptake

*SD,* standard deviation; *IQR,* interquartile range; *PET-CT;* positron emission tomography with computed tomography

*Serum Leucocytes and creatinine levels were measured as part of clinical practice ± 5 days after surgery and glucose levels were measured on the day of 18-FDG PET-CT scan

**Statistical difference between the three groups 1, 2 and 3

## Discussion

The present study showed lower ^18^F-FDG uptake in patients with the longest time between sternotomy and imaging compared to the other groups. Furthermore, in our qualitative assessment of uptake patterns we demonstrated a variety of ^18^F-FDG distribution patterns that can occur in healthy and non-infected patients after sternotomy with a tendency to normalize over time. Nevertheless, even in the group 3 with the longest time (55 ± 5 weeks) between imaging and sternotomy persistent elevated ^18^F-FDG uptake was observed in 45% of which 33% showed a persistent high-grade diffuse uptake pattern. All patients in this study were healthy and non-infected patients without suspicion of DSWI and therefore the uptake must be attributed to the operation and not to infection. As a consequence, ^18^F-FDG uptake on PET/CT until at least 1-year post-surgery must be interpreted with caution, especially in the early postoperative stage. To our knowledge this is the first study to evaluate prospectively the natural evolution of post-operative inflammatory changes at the sternotomy site. ^18^F-FDG PET/CT is known for high sensitivity in detection of infection but when used post-surgery the specificity is compromised. Physiological ^18^F-FDG uptake due to normal healing process, postoperative inflammation or incomplete ossification of the sternum could falsely be interpreted as pathological. Knowledge of normal ^18^F-FDG uptake distribution patterns is crucial in diagnosing suspected DSWI on ^18^F-FDG PET/CT imaging.

^18^F-FDG PET/CT has an established role in the detection of infection however after implantation of devices and prosthesis and in a post-surgical situation, the specificity is diminished.^8^ Literature on the ^18^F-FDG PET/CT assessment in cases of DSWI after sternotomy is scarce. The potential utility of ^18^F-FDG -PET/CT for diagnosing DSWI after sternotomy has been illustrated in a small case series^4, 5, 9^ One of these studies analyzed 73 patients with suspected DSWI retrospectively to analyze the accuracy of the^18^F-FDG -PET/CT. Sternal osteomyelitis showed a sensitivity and specificity of 98.4% and 77.8%, respectively (4). This study however lacked a control group, whereas 72/73 patients were diagnosed with sternal wound infection. Furthermore, imaging interpretation was done on uptake intensity cutoffs, however these cutoffs for DSWI are arbitrary and ^18^F-FDG distribution patterns were not taken into account. Another retrospective study with ^18^F-FDG -PET/CT imaging in confirmed cases of DSWI infection were compared with non-infected patients after sternotomy. This study emphasized the importance of ^18^F-FDG distribution pattern additional to ^18^F-FDG uptake levels and proposed a PET/CT imaging score which is solely based on qualitative imaging features.^5^ The area under the curve (AUC) of this scoring system is 0.96. However, the number of patients with confirmed DSWI in this study was limited (n = 11). Interestingly, the non-infected group (n = 29) in this study, with 42.4 months (mean) between sternotomy and imaging, showed diffuse high-grade uptake in 10/29 (34%) and low-grade uptake of 11/29 (38%). These results are comparable with our observed uptake patterns in group 3 with 3/20 (15%) diffuse high grade and 6/20 (30%) low-grade uptake.

The diagnostic accuracy of contrast enhanced CT also depends on timing of the CT examination. Mediastinal fluid collections, with or without free gas collection has been proposed as one of the primary imaging findings for DSWI. Nevertheless, the specificity in early stage postoperative (< 21 days) period only reaches 39%.^10^ Mediastinal fluid collection can also be detected on ^18^F-FDG -PET/CT as shown in our cohort. One retrospective study interestingly compared clinical outcome for DSWI between groups analyzed with CT and ^18^F-FDG -PET/CT. This study claims ^18^F-FDG -PET/CT is more accurate than CT in diagnosing and localizing DSWI after sternotomy, which leads to a more successful surgical debridement with lower recurrence rate (21.4% vs. 41.3%, *p* = 0.003) and shorter length of hospital stay (17.9 vs. 28.8 days, *p* < 0.001). This study might show benefit of ^18^F-FDG -PET/CT due to better localizing of the infection site compared to CT resulting in better clinical outcome. However only patients with suspected DSWI were included in the study and concerns regarding false positive diagnosis were not addressed.^11^

Magnetic resonance imaging (MRI) is useful for assessing chronic osteomyelitis however the use in patients with suspected DSWI its utility is limited due to motion artifacts and susceptibility artifacts because of metal sternal wires.^12^ Single-photon emission computed tomography (SPECT) for ^99m^Tc-hexamethylpropyleneamine oxime (HMPAO)-labeled leukocytes can also be used as imaging modality when DSWI is suspected after sternotomy and can differentiate between superficial and deep sternal wound infection. However, there is a delay in the diagnosis of 20 h between injection and imaging and there are concerns about the radioactivity.^13^. Like ^18^F-FDG -PET/CT this method also shows a variety of postoperative uptake patterns after sternotomy in healthy individuals.^14^ A recent study of 43 postoperative patients with suspected mediastinitis relapse showed an inconclusive result in 11.6% patients after 4 h and 20 h between imaging and injection. The authors claim a sensitivity of 100% but a 86% specificity and 85% positive predictive value. This study also had no control group.^15^

Our study has some limitations. First the laboratory results of serum levels of leucocytes were not analyzed prior to the imaging but were measured as part of clinical practice approximately 5 days after surgery. However, we included only healthy patients without suspicion of infection or DSWI. Secondly, imaging was performed once in every patient and not multiple times in the same patient. This approach was not deemed feasible due to the unjustified radiation dose of multiple ^18^F-FDG -PET/CT scans in individual healthy patients. Lastly, diabetes mellitus and obesity are condition that might affect the healing process following surgery. Our cohort did not include these patients and therefore additional analysis in these subgroups could not be performed.

## Conclusion

This study shows significant lower sternal ^18^F-FDG uptake in healthy (non-infected) patients at 55 ± 5 weeks after sternotomy compared to patients imaged within the first 3 months after surgery. However, even at 55 ± 5 weeks post sternotomy, elevated ^18^F-FDG uptake patterns at the sternum still persist in 45% of patients. Therefore, caution for false positive results must be taken into account when ^18^F-FDG -PET/CT is used in evaluation for DSWI during the first year after sternotomy. Further research must be done to establish the role of ^18^F-FDG -PET/CT in this diagnostic challenge.

## New knowledge gained

New knowledge is gained of the normal healing process and associated ^18^F-FDG uptake of non-infectious patients post-sternotomy to distinguish these patterns from pathological uptake.

## Supplementary Information

Below is the link to the electronic supplementary material.

Supplementary file1 (PPTX 448 KB)

Supplementary file2 (MP3 4763 KB)

## Abbreviations

DSWI: Deep Sternum Wound Infection
^18^F-FDG: ^18^F-fluorodeoxyglucose
PET: Positron Emission Tomography
CT: Computed Tomography
^18^F-FDG PET/CT: ^18^F-fluorodeoxyglucose Positron Emission Tomography/Computed Tomography
AVR: Aortic valve replacement
SUVmax: Maximum Standardized Uptake Value
SUVmean: Mean Standardized Uptake Value
