# Supplementary material for: 18F-FDG/PET-CT imaging findings after sternotomy
Source: J Nucl Cardiol. 2022 Nov 8;30(3):1210–8. doi: 10.1007/s12350-022-03126-x (PMC10261398; doi:10.1007/s12350-022-03126-x)
Supplement: Supplementary file 1 — Supplementary file1 (PPTX 448 KB) [file 12350_2022_3126_MOESM1_ESM.pptx]

## Slide 1
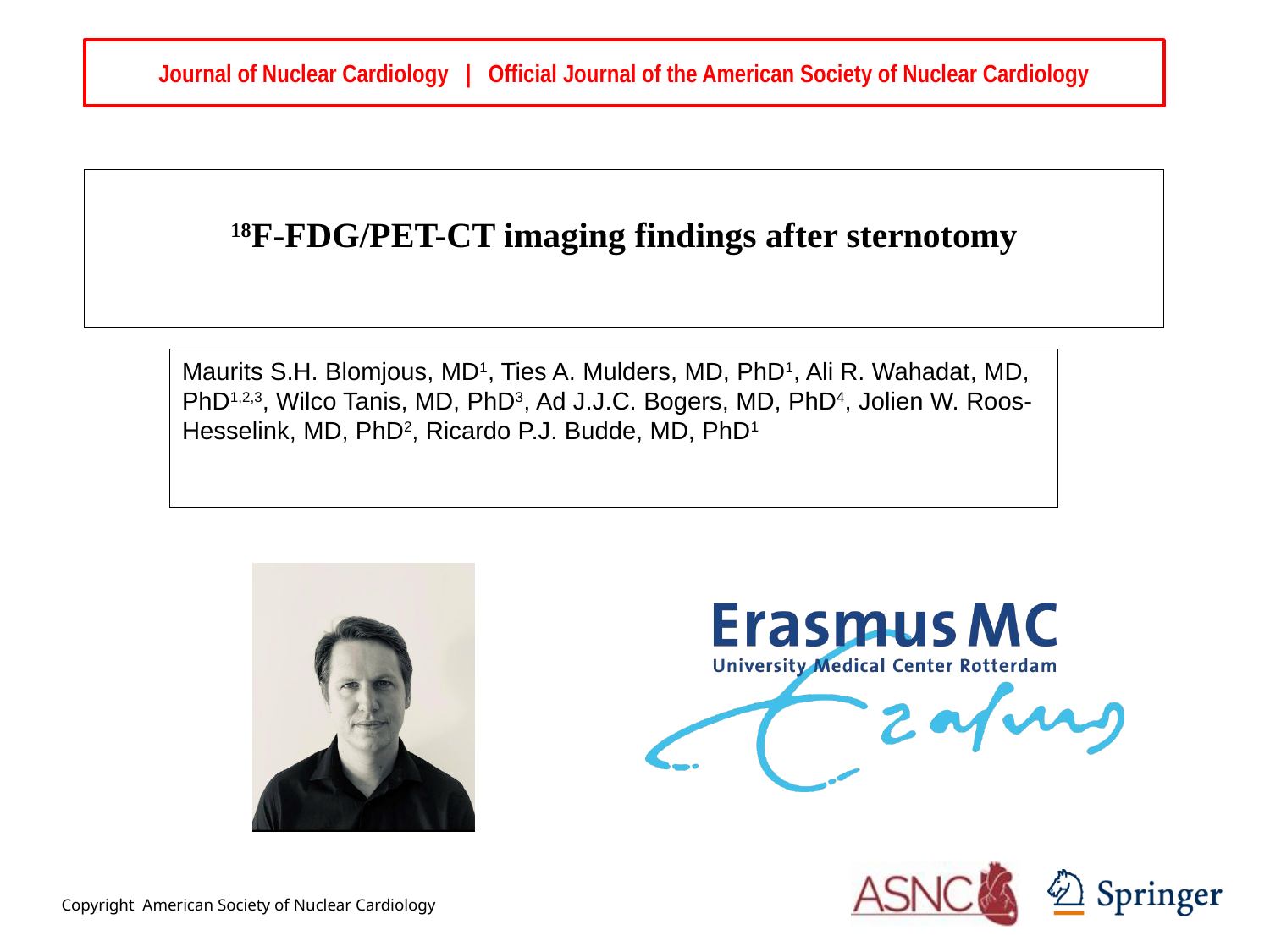

Journal of Nuclear Cardiology | Official Journal of the American Society of Nuclear Cardiology
# 18F-FDG/PET-CT imaging findings after sternotomy
Maurits S.H. Blomjous, MD1, Ties A. Mulders, MD, PhD1, Ali R. Wahadat, MD, PhD1,2,3, Wilco Tanis, MD, PhD3, Ad J.J.C. Bogers, MD, PhD4, Jolien W. Roos-Hesselink, MD, PhD2, Ricardo P.J. Budde, MD, PhD1
Copyright American Society of Nuclear Cardiology

## Slide 2
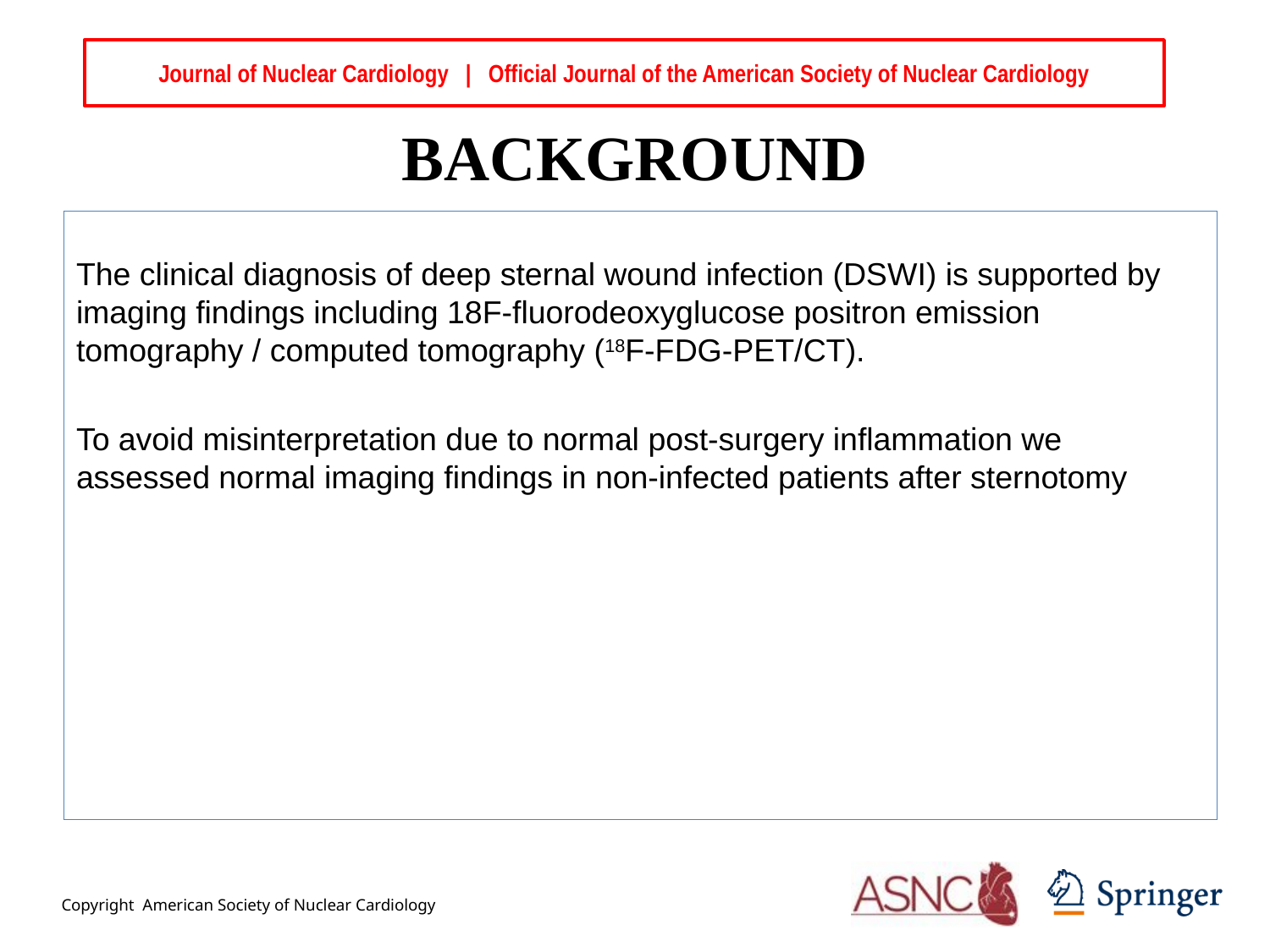

Journal of Nuclear Cardiology | Official Journal of the American Society of Nuclear Cardiology
# BACKGROUND
The clinical diagnosis of deep sternal wound infection (DSWI) is supported by imaging findings including 18F-fluorodeoxyglucose positron emission tomography / computed tomography (18F-FDG-PET/CT).
To avoid misinterpretation due to normal post-surgery inflammation we assessed normal imaging findings in non-infected patients after sternotomy
Copyright American Society of Nuclear Cardiology

## Slide 3
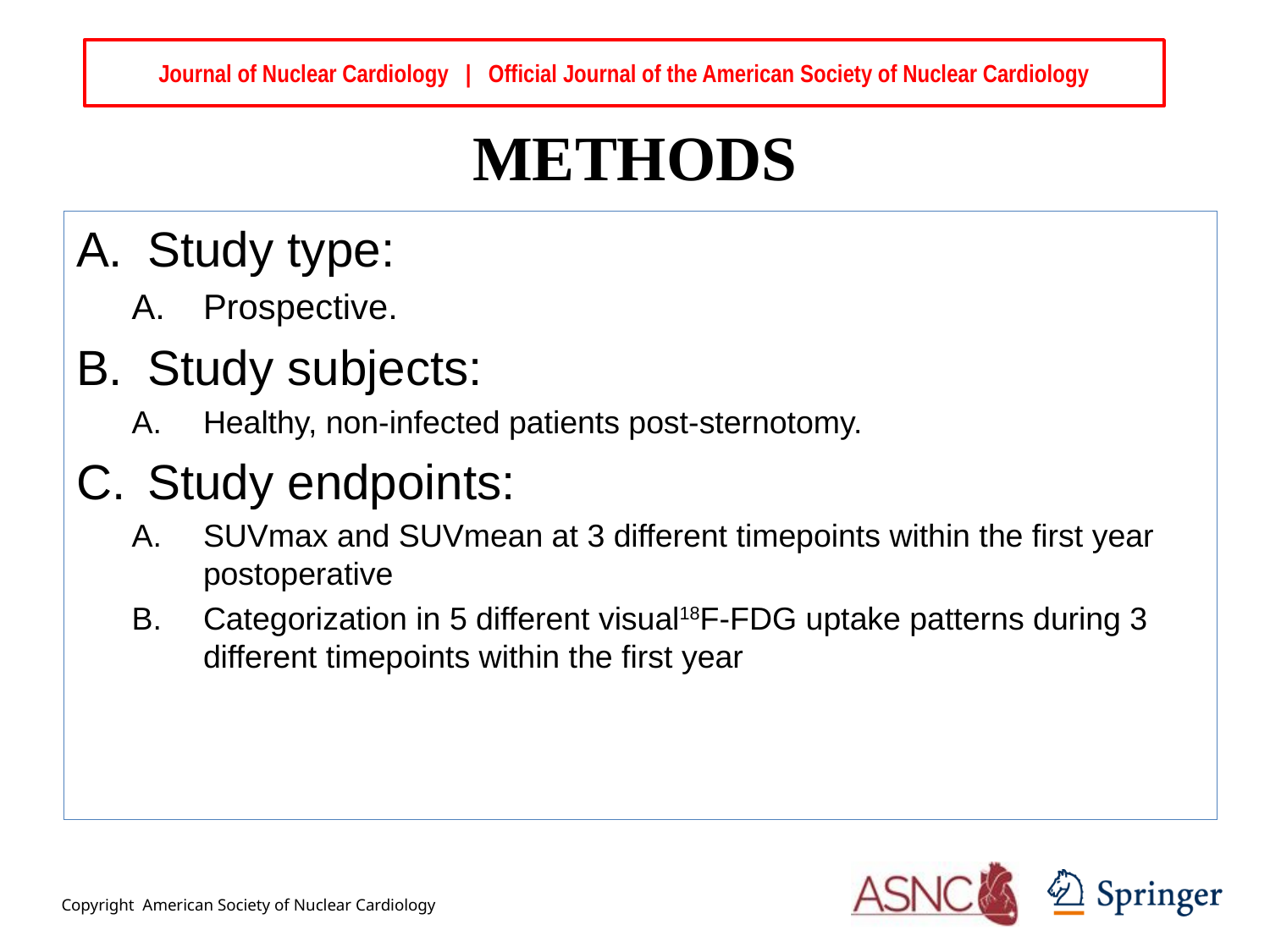

Journal of Nuclear Cardiology | Official Journal of the American Society of Nuclear Cardiology
# METHODS
Study type:
Prospective.
Study subjects:
Healthy, non-infected patients post-sternotomy.
Study endpoints:
SUVmax and SUVmean at 3 different timepoints within the first year postoperative
Categorization in 5 different visual18F-FDG uptake patterns during 3 different timepoints within the first year
Copyright American Society of Nuclear Cardiology

## Slide 4
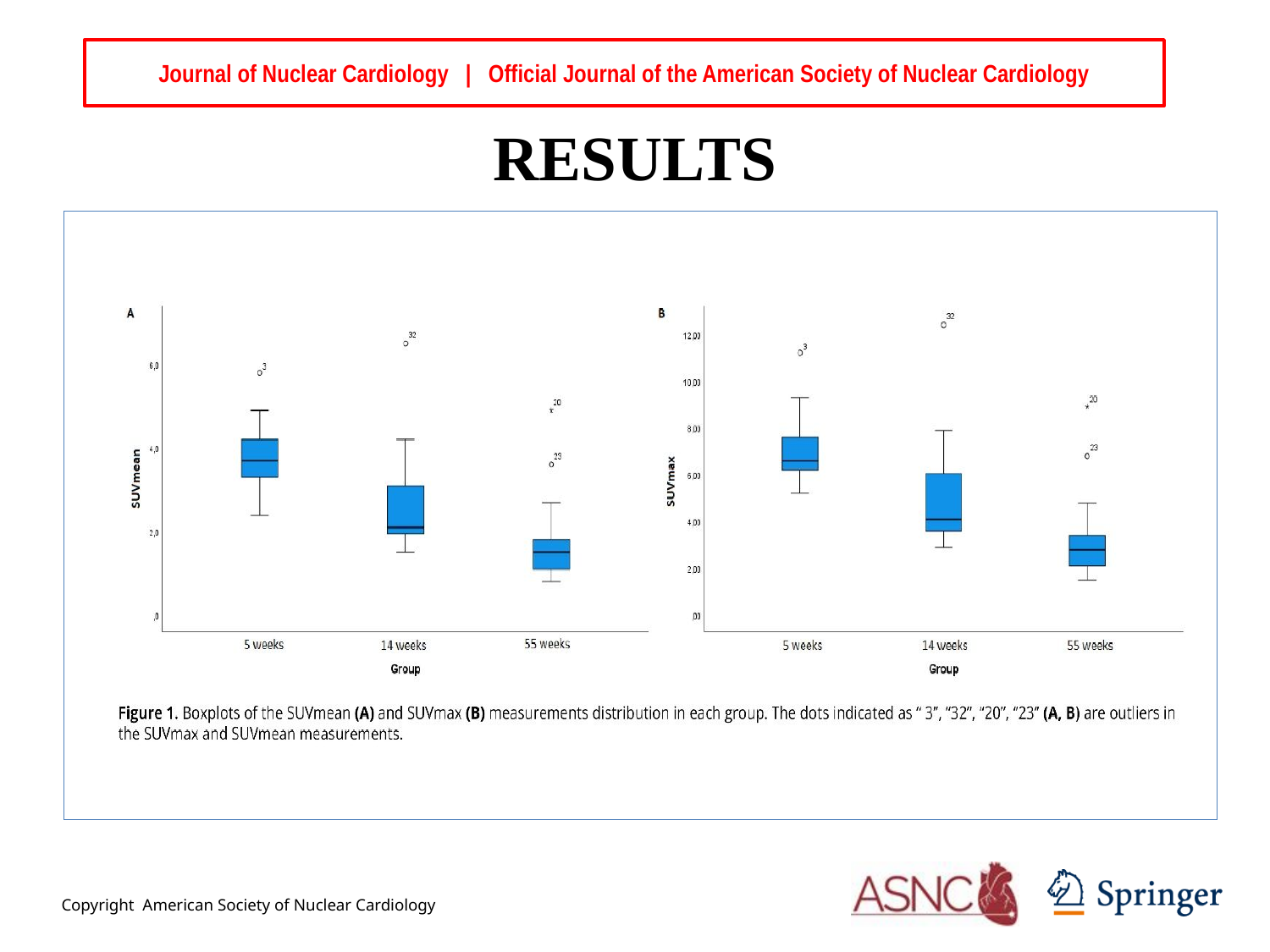

Journal of Nuclear Cardiology | Official Journal of the American Society of Nuclear Cardiology
# RESULTS
Insert a key table or a key figure
If figure, insert legend
Copyright American Society of Nuclear Cardiology

## Slide 5
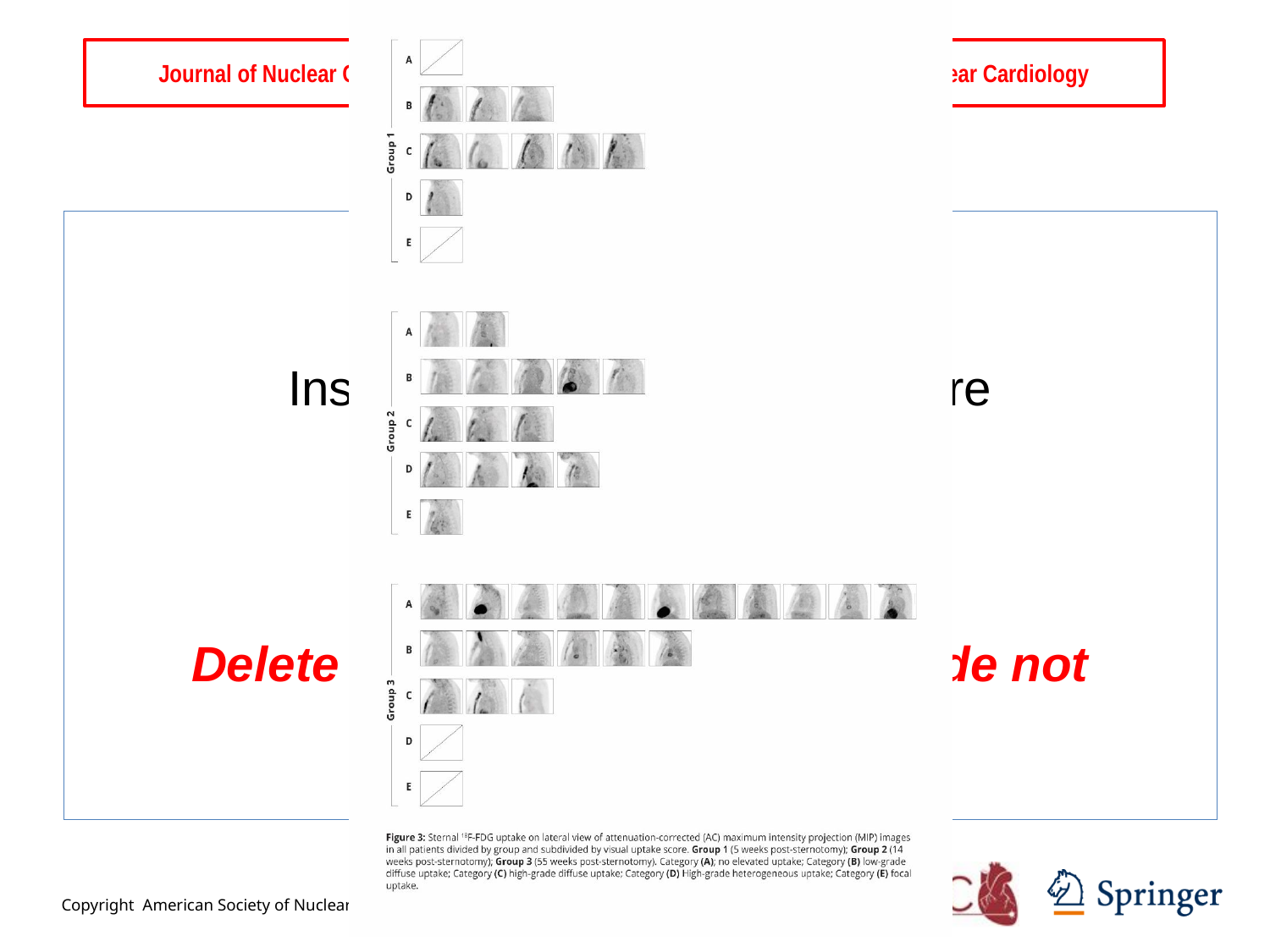

Journal of Nuclear Cardiology | Official Journal of the American Society of Nuclear Cardiology
# RESULTS
Insert a key table or a key figure
If figure, insert legend
Delete slide if second results slide not necessary
Copyright American Society of Nuclear Cardiology

## Slide 6
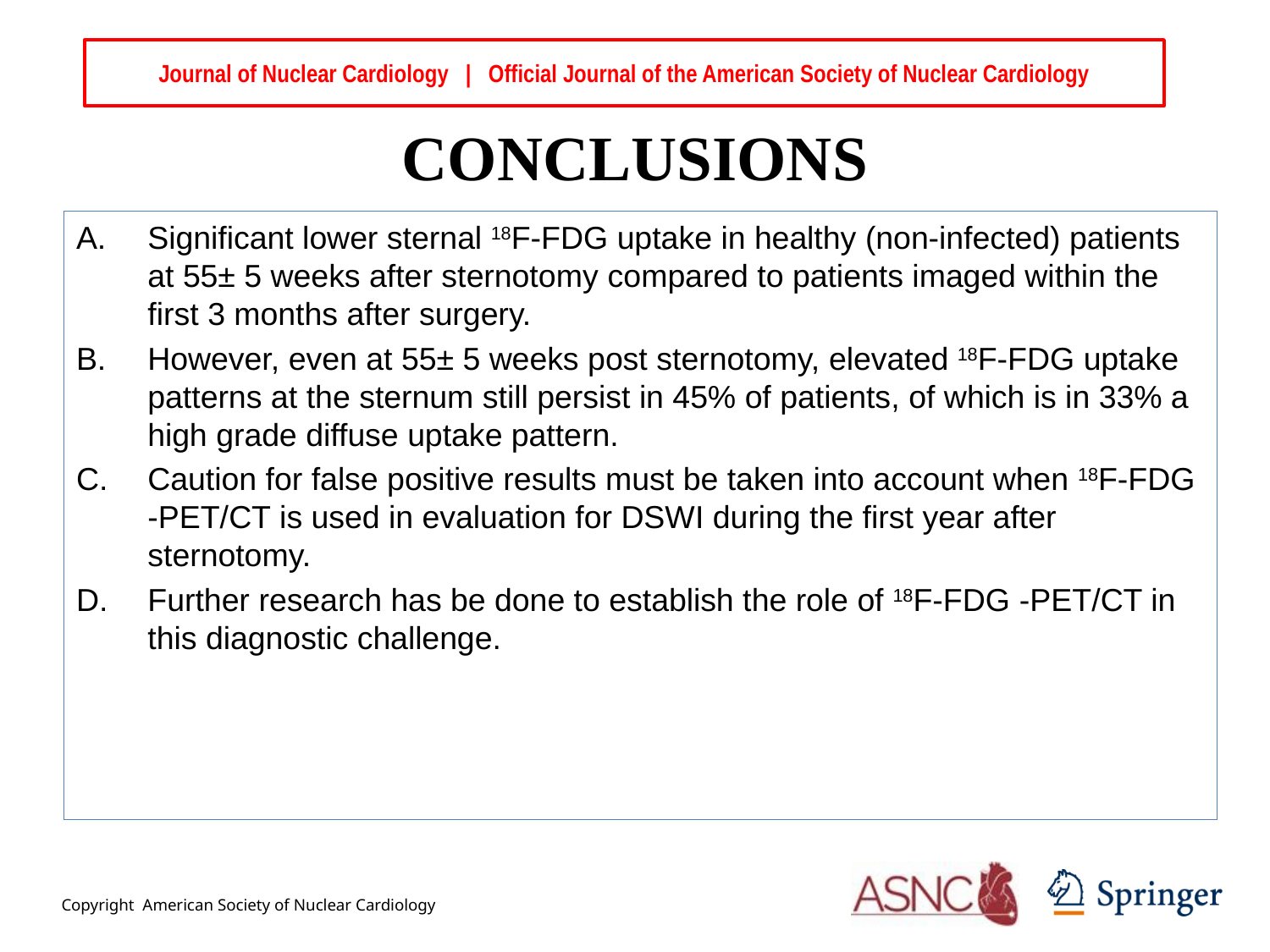

Journal of Nuclear Cardiology | Official Journal of the American Society of Nuclear Cardiology
# CONCLUSIONS
Significant lower sternal 18F-FDG uptake in healthy (non-infected) patients at 55± 5 weeks after sternotomy compared to patients imaged within the first 3 months after surgery.
However, even at 55± 5 weeks post sternotomy, elevated 18F-FDG uptake patterns at the sternum still persist in 45% of patients, of which is in 33% a high grade diffuse uptake pattern.
Caution for false positive results must be taken into account when 18F-FDG -PET/CT is used in evaluation for DSWI during the first year after sternotomy.
Further research has be done to establish the role of 18F-FDG -PET/CT in this diagnostic challenge.
Copyright American Society of Nuclear Cardiology
